# Books

**Published:** 1883-08

**Authors:** 


### Books.

The Microscope and Its Revelations, by Wm. B Carpenter, C. B., M.D., L.L.D., has been laid upon our table, but too late for such a notice as the merits of the book require. It shall receive due respect in our next number. Also, the fifteenth volume of the Transactions of the Medical Society of Pennsylvania. This is quite a bulky volume and contains some valuable contributions to medical literature, which we will reproduce as opportunity permits.

Infantal Paralysis.—Dr. Barlow thinks that electricity is the chief agent to be relied on in this trouble. The earlier the treatment is begun the better. Stimulate the muscle first with the constant and later on with the induced current. At the same time have the patient attempt to move the affected parts.—*British Med. Journal.*

